# STAR: predicting recombination sites from amino acid sequence

**DOI:** 10.1186/1471-2105-7-437

**Published:** 2006-10-08

**Authors:** Denis C Bauer, Mikael Bodén, Ricarda Thier, Elizabeth M Gillam

**Affiliations:** 1 Institute for Molecular Bioscience, The University of Queensland, QLD 4072, Australia; 2 School of Information Technology and Electrical Engineering, The University of Queensland, QLD 4072, Australia; 3 School of Biomedical Sciences, The University of Queensland, QLD 4072, Australia

## Abstract

**Background:**

Designing novel proteins with site-directed recombination has enormous prospects. By locating effective recombination sites for swapping sequence parts, the probability that hybrid sequences have the desired properties is increased dramatically. The prohibitive requirements for applying current tools led us to investigate machine learning to assist in finding useful recombination sites from amino acid sequence alone.

**Results:**

We present STAR, Site Targeted Amino acid Recombination predictor, which produces a score indicating the structural disruption caused by recombination, for each position in an amino acid sequence. Example predictions contrasted with those of alternative tools, illustrate STAR'S utility to assist in determining useful recombination sites. Overall, the correlation coefficient between the output of the experimentally validated protein design algorithm SCHEMA and the prediction of STAR is very high (0.89).

**Conclusion:**

STAR allows the user to explore useful recombination sites in amino acid sequences with unknown structure and unknown evolutionary origin. The predictor service is available from .

## Background

Recombinant DNA techniques, such as DNA shuffling, generate more diverse libraries than random mutagenesis with a relatively high fraction of functional proteins [1-4]. Like mutagenesis but unlike *de novo *protein design, recombination deals with native sequences whose effectiveness for some particular function is established. As protein design tools, recombinatorial techniques dramatically reduce the combinatorial space of possible sequences to an area which can be explored *in vitro *more easily.

Site-directed (as opposed to random) recombination systematically reduces the amino acid sequence space for consideration by identifying specific sites in parental sequences at which their parts can be interchanged [2]. This paper develops and evaluates a method that uses machine learning to suggest recombination sites solely from the amino acid sequences of the parents. Alternative tools may be more precise but require either parents to be structurally resolved [5] or phylogenetically well-characterised [6].

SCHEMA predicts structural disruption by gleaning contact maps of parents [5]. The principle of SCHEMA applies to recombination site identification (using SCHEMA's so-called S-profile) or to evaluate the folding potential of specific hybrids (using SCHEMA's E-value). RASPP (Recombination as a Shortest-Path Problem) was introduced to generate and evaluate candidate hybrids using the E-value [7].

SCHEMA's S-profile essentially identifies sequence segments that are likely to fold independently from the rest of the protein [5]. The profile is the series of sums of possible disruptions (caused by recombination) within a window centered on each residue of the protein (see Equation 1). The assumption behind SCHEMA is that protein function is preserved by not interfering with these structural "building blocks". Different segments are sampled from a family of parent proteins to be recombined with the main structural features left intact [5]. Indeed, sites identified by SCHEMA match successful recombination sites used in *in vitro *experiments [5]. Additionally, the application of SCHEMA on the *β*-lactamase and cytochrome P450 families has been helpful in evaluating (and maximising) the ratio of folded and functional enzyme hybrids [3,8]. Note, SCHEMA's "building blocks" must not be confused with domains or other structural or functional subdivisions (motifs, modules or exons). By placing recombination sites only at the boundaries of such structural subdivisions, exploration is severely hampered [2]. Additionally, to enhance a function, intra-domain segments may need to be perturbed.

Unfortunately, SCHEMA requires the full tertiary description of the protein structure (as in the Protein Data Bank, PDB). This requirement severely limits the number of candidate proteins to the small group that are already resolved. Due to the expensive, time-consuming and complicated nature of structure determination, the number of proteins with known structure is likely to remain comparably small to the number of known sequences.

FamClash is another method for evaluating the potency of hybrids [6]. FamClash checks for every amino acid pair [*i*, *j*] (residue at position *i *in parent 1 and position *j *in parent 2) if charge, volume and hydrophobicity is in agreement with the conserved properties at [*i*, *j*] in the protein family both parents belong to. To determine the conserved properties of the family a preprocessing step is necessary. Each residue pair in the *m*th member of the family forms a unique 3D coordinate [Ci,jm
 MathType@MTEF@5@5@+=feaafiart1ev1aaatCvAUfKttLearuWrP9MDH5MBPbIqV92AaeXatLxBI9gBaebbnrfifHhDYfgasaacH8akY=wiFfYdH8Gipec8Eeeu0xXdbba9frFj0=OqFfea0dXdd9vqai=hGuQ8kuc9pgc9s8qqaq=dirpe0xb9q8qiLsFr0=vr0=vr0dc8meaabaqaciaacaGaaeqabaqabeGadaaakeaacqWGdbWqdaqhaaWcbaGaemyAaKMaeiilaWIaemOAaOgabaGaemyBa0gaaaaa@32E3@, Vi,jm
 MathType@MTEF@5@5@+=feaafiart1ev1aaatCvAUfKttLearuWrP9MDH5MBPbIqV92AaeXatLxBI9gBaebbnrfifHhDYfgasaacH8akY=wiFfYdH8Gipec8Eeeu0xXdbba9frFj0=OqFfea0dXdd9vqai=hGuQ8kuc9pgc9s8qqaq=dirpe0xb9q8qiLsFr0=vr0=vr0dc8meaabaqaciaacaGaaeqabaqabeGadaaakeaacqWGwbGvdaqhaaWcbaGaemyAaKMaeiilaWIaemOAaOgabaGaemyBa0gaaaaa@3309@, Hi,jm
 MathType@MTEF@5@5@+=feaafiart1ev1aaatCvAUfKttLearuWrP9MDH5MBPbIqV92AaeXatLxBI9gBaebbnrfifHhDYfgasaacH8akY=wiFfYdH8Gipec8Eeeu0xXdbba9frFj0=OqFfea0dXdd9vqai=hGuQ8kuc9pgc9s8qqaq=dirpe0xb9q8qiLsFr0=vr0=vr0dc8meaabaqaciaacaGaaeqabaqabeGadaaakeaacqWGibasdaqhaaWcbaGaemyAaKMaeiilaWIaemOAaOgabaGaemyBa0gaaaaa@32ED@] with Ci,jm
 MathType@MTEF@5@5@+=feaafiart1ev1aaatCvAUfKttLearuWrP9MDH5MBPbIqV92AaeXatLxBI9gBaebbnrfifHhDYfgasaacH8akY=wiFfYdH8Gipec8Eeeu0xXdbba9frFj0=OqFfea0dXdd9vqai=hGuQ8kuc9pgc9s8qqaq=dirpe0xb9q8qiLsFr0=vr0=vr0dc8meaabaqaciaacaGaaeqabaqabeGadaaakeaacqWGdbWqdaqhaaWcbaGaemyAaKMaeiilaWIaemOAaOgabaGaemyBa0gaaaaa@32E3@ = *C*(*k*) + *C*(*l*), where *k *is the residue at position *i *in *m *and *l *at *j *and *C*(·) is the value of a lookup table containing the charge of the residue (*V *is the volume and *H *the hydrophobicity, respectively). The space is partitioned into cubes. Residue pairs whose coordinates are within one cube are defined to be similar. A position [*i*, *j*] is conserved if 20% of the family members have a residue pair for [*i*, *j*] that is within the cube. Each residue pair in the hybrid can now be assessed with the triplet of the mean values of the conserved residue pairs in the protein family. Any deviation between hybrid and conserved property in the family is denoted as a "clash". Positions with no conservation are simply ignored. A limitation of FamClash is its reliance on members of the parents' protein family. A sufficiently large protein family needs to be established and analysed to properly identify conservation.

Saraf et al. developed OPTCOMB [9], which like RASPP identifies the optimal recombination sites and, additionally, is able to limit the parental sequence fragments for the library to the most promising ones. FamClash [6] is used as an objective function. Thus, OPTCOMB identifies recombination sites which produce hybrids with the minimal number of clashes. Like RASPP, OPTCOMB is able to use any function that evaluates a protein including SCHEMA [9] or the current method.

Using machine learning methods, we developed STAR (Site Targeted Amino acid Recombination predictor), to extend a SCHEMA-like analysis to proteins for which no structure has been solved. STAR predicts the maximum number of connections that can be broken by recombination for each position in the parent – SCHEMA's single-parent S-score (see Equation 1). A minimum in this STAR-profile corresponds to a region where the protein structure has lower contact density and thus less prone to be corrupted by recombination.

Notably, SCHEMA's S-score has largely been superseded by the E-value (which is now the recommended choice according to the original SCHEMA authors). Since the intention here is partly to explore the applicability of machine learning tools to assist in the determination of recombination sites, we choose to focus on the simpler strategy based on the S-score as calculated from a single sequence.

For comparison, I-Mutant2.0 [10], MUpro [11] and Conseq [12] predict stability changes caused by single-point mutation from amino acid sequence data. These methods are not specifically designed to assist in the determination of recombination sites.

However, by exhaustively testing all possible amino acid substitution for each position, we can identify crucial amino acids within the sequence, and compare these scores with STAR's. I-Mutant2.0 and MUpro are both machine learning-based and predict a stability change (positive or negative) of the whole protein for a given single-point mutation. If a residue is crucial for retaining the current structure, substitutions would result in large negative predictions. Conseq uses phylogenetic trees to calculate a conservation score for each residue which should correlate with the importance of it.

## Implementation

The goal is to develop a model using machine learning methods to predict SCHEMA's *single-*parent S-profile from an amino acid sequence. From a machine learning point of view, the goal presents a significant challenge since SCHEMA relies crucially on structural features, not amino acid composition. As per Equation 1 the S-score counts the number of connections that break if position *i *serves as the point at which sequence parts are exchanged.

Si=∑j=i−w+1i∑k=jj+w−2∑l=k+1j+w−1ckl     (1)
 MathType@MTEF@5@5@+=feaafiart1ev1aaatCvAUfKttLearuWrP9MDH5MBPbIqV92AaeXatLxBI9gBaebbnrfifHhDYfgasaacH8akY=wiFfYdH8Gipec8Eeeu0xXdbba9frFj0=OqFfea0dXdd9vqai=hGuQ8kuc9pgc9s8qqaq=dirpe0xb9q8qiLsFr0=vr0=vr0dc8meaabaqaciaacaGaaeqabaqabeGadaaakeaacqWGtbWudaWgaaWcbaGaemyAaKgabeaakiabg2da9maaqahabaWaaabCaeaadaaeWbqaaiabdogaJnaaBaaaleaacqWGRbWAcqWGSbaBaeqaaaqaaiabdYgaSjabg2da9iabdUgaRjabgUcaRiabigdaXaqaaiabdQgaQjabgUcaRiabdEha3jabgkHiTiabigdaXaqdcqGHris5aaWcbaGaem4AaSMaeyypa0JaemOAaOgabaGaemOAaOMaey4kaSIaem4DaCNaeyOeI0IaeGOmaidaniabggHiLdaaleaacqWGQbGAcqGH9aqpcqWGPbqAcqGHsislcqWG3bWDcqGHRaWkcqaIXaqmaeaacqWGPbqAa0GaeyyeIuoakiaaxMaacaWLjaWaaeWaaeaacqaIXaqmaiaawIcacaGLPaaaaaa@5D00@

*S_i _*describes, for each sequence position *i*, the number of contacts within the window (*i *- *w*, *i *+ *w*) that could be broken if the recombination site is positioned at *i*. *c*_*kl *_= 1 if residues *k *and *l *are in within 4.5 Å of one another, and *c*_*kl *_= 0 otherwise. In the *multi-*parent S-score, *c_kl _*is derived from a multiply-aligned contact map [5]. *c_kl _*only counts when the two residues *k *and *I *come from different parents and represent different amino acids. The single-parent S-score neglects the overlap between parents and is thus an upper bound of the multi-parent S-score (and converges to it with decreasing parent sequence similarity). To follow the configuration used by [5], *w *is set to 14 residues.

To build a data set for training and evaluating machine learning models, the binary contact map was derived for each of the proteins in a 945-protein data set (extracted from PDB) by employing a Euclidian cut-off distance of 4.5 Å. Equation 1 is then determined for each residue in the set. For practical purposes, the single-parent S-score is normalised to fall in the 0–1 interval: SiN
 MathType@MTEF@5@5@+=feaafiart1ev1aaatCvAUfKttLearuWrP9MDH5MBPbIqV92AaeXatLxBI9gBaebbnrfifHhDYfgasaacH8akY=wiFfYdH8Gipec8Eeeu0xXdbba9frFj0=OqFfea0dXdd9vqai=hGuQ8kuc9pgc9s8qqaq=dirpe0xb9q8qiLsFr0=vr0=vr0dc8meaabaqaciaacaGaaeqabaqabeGadaaakeaacqWGtbWudaqhaaWcbaGaemyAaKgabaGaemOta4eaaaaa@3088@ = *tanh*(*S*_*i*_/*max*) where we have preset *max *= 637 from gleaning the data set. We call this score the *calculated *STAR-score. The sequence set represents a diverse range of proteins and has no pairs with more than 25% sequence similarity [13]. The data set is made available from the predictor home page.

## Model development

PSI-BLAST profiles are used successfully for most protein structure prediction problems, including secondary structure, residue contacts, and surface accessibility [14,15]. In a standard fashion, each protein chain in the data set is represented using the PSI-BLAST profile (PSI-BLAST is run with 3 iterations over Genbank's non-redundant protein set). The profile implicitly incorporates information about sequence variability and the location of indels within a family of proteins [14].

We evaluate two major types of machine learning algorithms, namely Support Vector Regression and Neural Networks. Both types have repeatedly been found superior for relevant prediction problems (e.g. secondary structure prediction [14,16,17], contact number and solvent accessibility prediction [15]). The input window size of all models is set to 15 residues: the residue for which the SCHEMA score is predicted and then 7+7 residues immediately upstream and downstream, respectively. We use 2-fold crossvalidation to develop and test models, meaning that one simulation run involves training and testing two models. Each model is trained on half the data and tested on the remaining half but controlled so that each data point appears as a test point in exactly one model. Preliminary simulations with the used models showed that the differences between 10-fold and 2-fold crossvalidation are negligible (not shown). To minimize the computational time required, we consistently use 2-fold crossvalidation to develop and test the models. However, we repeated the runs to guarantee consistency in the results. The average accuracy is reported below.

The STAR-score is not predicted with a reasonable accuracy if the machine learning algorithm is presented with only the sequence data encoded using PSI-BLAST profiles (see Table 1). Instead we first present the sequence to an already existing secondary structure predictor and then use its output as the input to our STAR-score predictor. To predict the secondary structure, we use the Continuum Secondary Structure Predictor [17] which produces a probability for each secondary structure state. Noteworthy, the Continuum Secondary Structure has a state-of-the-art classification accuracy of *Q*_3 _= 77.3 and from preliminary trials we note there is no significant difference in STAR-score prediction accuracy if the true secondary structure is used as input instead.

**Table 1:** The STAR-score prediction accuracy.

Configuration	r
FFNN (0 hidden)	0.56
FFNN (20 hidden)	0.56
FFNN (40 hidden)	0.56
BRNN (7+7 hidden)	0.66

The STAR-score prediction accuracy (established from test data using the correlation with the *calculated *STAR-score) when sequence data was presented directly to the machine learning algorithm.

The simple Feed Forward Neural Network (FFNN) and the Bidirectional Recurrent Neural Network (BRNN) [18] are trained and evaluated on the 945-protein data set. Training uses gradient descent to minimise the error as measured on the single output node. The learning rate is *η *= 0.001. A variety of hidden node numbers *h *(including not using a hidden layer at all) are trialled. For all neural networks, training data is presented in batches of 100 windows before the weights are changed. A total of 40,000 sequences were presented in random order before we stopped training. In preliminary studies this number was seen as sufficient for convergence.

Recent findings suggest that Support Vector Regression (SVR) exceeds the accuracy reached by many neural networks [19,20]. Essentially, support vector regression operates by finding so-called support vectors that collectively represent the function in a feature space. A kernel function maps the input sequence encoding into the feature space. Support vector regression can be understood as minimising a tube wrapped around the hypothesis function. During training, the "tube" is defined by an *ε*-insensitive loss function (where *ε *represents the size of a margin to the hypothesis function). Sample targets outside this margin are penalised. For *ε*, we use the standard value of 0.1. We examine optimisation using *ε*-SVR and *ν*-SVR with the same protein data set. *ν*-SVR replaces the *ε *hyper-parameter with *ν*, to control the number of support vectors (*ν *= 0.5 in all simulations). The standard stopping criterion is used and *C *was set to 0.5 (which delivered a better result than the standard value of 1). We trial both the Linear and Gaussian kernel functions (with *γ *= 1). Note, the exploration of the parameter space is far from being exhaustive.

We use the correlation coefficient *r *between the calculated STAR-score *t_i _*and the predicted score *p_i _*where the index *i *represents the position in the sequence, *r *is defined for a single chain.

r=〈(ti−〈ti〉)⋅(pi−〈pi〉)〉〈(ti−〈ti〉)〉⋅〈(pi−〈pi〉)〉     (2)
 MathType@MTEF@5@5@+=feaafiart1ev1aaatCvAUfKttLearuWrP9MDH5MBPbIqV92AaeXatLxBI9gBaebbnrfifHhDYfgasaacH8akY=wiFfYdH8Gipec8Eeeu0xXdbba9frFj0=OqFfea0dXdd9vqai=hGuQ8kuc9pgc9s8qqaq=dirpe0xb9q8qiLsFr0=vr0=vr0dc8meaabaqaciaacaGaaeqabaqabeGadaaakeaacqWGYbGCcqGH9aqpdaWcaaqaaiabgMYiHlabcIcaOiabdsha0naaBaaaleaacqWGPbqAaeqaaOGaeyOeI0IaeyykJeUaemiDaq3aaSbaaSqaaiabdMgaPbqabaGccqGHQms8cqGGPaqkcqGHflY1cqGGOaakcqWGWbaCdaWgaaWcbaGaemyAaKgabeaakiabgkHiTiabgMYiHlabdchaWnaaBaaaleaacqWGPbqAaeqaaOGaeyOkJeVaeiykaKIaeyOkJepabaWaaOaaaeaacqGHPms4cqGGOaakcqWG0baDdaWgaaWcbaGaemyAaKgabeaakiabgkHiTiabgMYiHlabdsha0naaBaaaleaacqWGPbqAaeqaaOGaeyOkJeVaeiykaKIaeyOkJepaleqaaOGaeyyXIC9aaOaaaeaacqGHPms4cqGGOaakcqWGWbaCdaWgaaWcbaGaemyAaKgabeaakiabgkHiTiabgMYiHlabdchaWnaaBaaaleaacqWGPbqAaeqaaOGaeyOkJeVaeiykaKIaeyOkJepaleqaaaaakiaaxMaacaWLjaWaaeWaaeaacqaIYaGmaiaawIcacaGLPaaaaaa@72CD@

where ⟨·⟩ is the mean. Ideal performance means that *t_i _*and *p_i _*are perfectly and positively correlated *r *= 1 All reported result values are averages over all chains (when they appear as test cases).

## Results and discussion

The results when using the predicted secondary structure as input are shown in Table 2. BRNN seems to perform slightly better than the other algorithms but the small number of trials prohibits us from ranking them confidently.

**Table 2:** The STAR-score prediction accuracy.

Configuration	r
FFNN (0 hidden)	0.86
FFNN (20 hidden)	0.86
FFNN (40 hidden)	0.86
BRNN (7+7 hidden)	0.89
*ε*-SVR (Linear)	0.82
*ε*-SVR (Gaussian)	0.80
*ν*-SVR (Gaussian)	0.83

The STAR-score prediction accuracy (established from test data using the correlation with the *calculated *STAR-score) when sequence data was presented as the predicted 3-class Continuum Secondary Structure.

The average correlation coefficient between the predicted and calculated STAR-score for BRNN on test data (set aside from the 945-protein set using cross-validation) is 0.89 (1.0 represents perfect agreement, and 0.0 represents random agreement) and the mean squared error is 0.0028. Approximately 18% of the sequences from the STAR data set have a sequence similarity exceeding 25% with sequences in the training dataset of the Secondary Structure Predictor [21]. To ensure that the overlap does not influence the estimated accuracy, we assessed the STAR prediction accuracy specifically for the sequences which had less than 25% similarity with the set used for training the secondary structure predictor. The average correlation coefficient on these 771 was 0.88 (average mean squared error is 0.0028).

The most essential piece of information in the S-profile is the positions of minima [5]. In addition to the correlation, the distance between the positions of the minima in the predicted function and in the target function, illustrates the suitability of the method for protein design. The average distance between the position of the predicted and the target minima is a mere 3.42 residues for the BRNN-model.

The high correlation between the scores and the low average distance between their minimas, illustrate the feasibility of replacing the S-score with STAR – allowing the user to explore structural building blocks *in silica *of yet unresolved or even hypothetical protein with little loss of precision.

The web interface of STAR requires the user to input a single amino acid sequence (in the FASTA format), and then predicts the single-parent SCHEMA profile. The profile can be presented as a graph – allowing the user to quickly assess regions of low connectivity lending themselves to recombination. The individual scores are also presented numerically with added details (including the continuum secondary structure). A suitable recombination site is thus one with a low score, i.e. a position which – should it be the point of exchange – disrupts few, predicted connections in the parent sequence.

**Figure 1:**
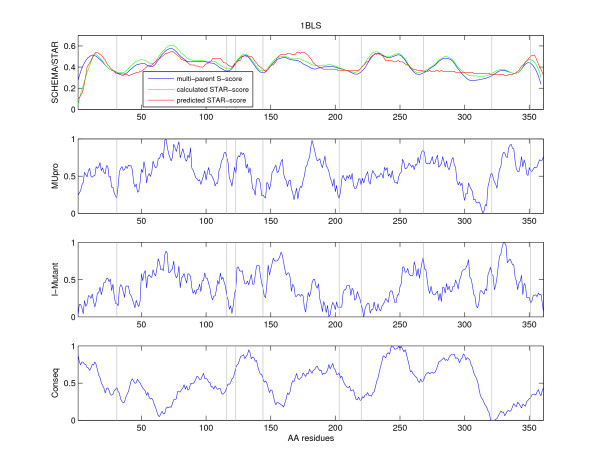
**The SCHEMA-profile, the STAR-profile, and post-processed profiles for Conseq, MUpro and l-Mutant2.0 for the protein **1BLS. (a) The multi-parent S-scores (normalised) along with the calculated and predicted STAR-scores for 1BLS. The profiles indicate the number of disrupted connections (y-axis) at each sequence position (x-axis). (b) The post-processed score from MUpro for 1BLS. The profile indicates the structural stability change caused by mutation (y-axis) for each sequence residue (x-axis). (c) The post-processed score from I-Mutant2.0 for 1BLS. The profile indicates the structural stability change caused by mutation (y-axis) for each sequence residue (x-axis). (d) The post-processed score from Conseq. The profile indicates the level of amino acid conservation (y-axis) for each sequence residue (x-axis). The successful recombination sites from a random DNA shuffling experiment are added to each graph and plotted as vertical lines [1].

A family of Cephalosporinase genes from four microbial species subjected to random DNA shuffling yielded an eight-fold increase of moxalactamase activity in a single cycle of shuffling [1]. We presented the sequence of PDB entry 1BLS (one of the four Cephalosporinase used in the original study) to STAR. We calculated the single-parent S-score (the calculated STAR-score) from the contact map of 1BLS and the multi-parent S-score using a sequence alignment between 1BLS and 1G68 (also used in the original study, exhibiting 40% sequence identity with 1BLS). Both SCHEMA-profiles were superimposed on top of the predicted STAR-score (see Figure 1). We also present the successful recombination sites as determined from the original random shuffling experiment. The same data was used for evaluating the original SCHEMA algorithm (cf. [5]). For practical reasons we were unable to test FamClash (but cf. [22])

**Figure 2:**
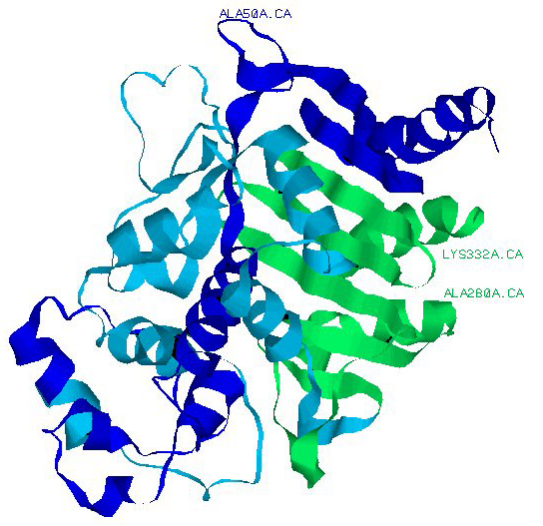
**The **1BLS**protein structure. **Three residues are identified, each translating into divergent SCHEMA- and STAR-scores.

As can be seen in Figure 1, the successful recombination sites match the minima of all profiles with the exception of the site close to the C-terminus. The last site is deemed unsuitable by both the original SCHEMA algorithm and STAR. The full structure shown in Figure 2 illustrates that regions which have divergent SCHEMA- and STAR-scores are at the surface of the molecule and should typically have lower connectivity (a generalisation that STAR seems to use).

To put the example prediction in context, we adapted alternative methods not explicitly designed for this purpose but potentially useful for identifying recombination sites. The predicted stability change of I-Mutant2.0 and MUpro is summed up over all 19 possible amino acid substitution for each position of the amino acid sequence. The lower the score for an amino acid, the more sensitive the current structure is to perturbations. The score profile is smoothed with a kernel averaging over 10 neighboring residues, normalised and inverted to compare neatly with STAR's profile. This post-processed prediction thus has minima at positions with high substitution tolerance. Conseq predicts a conservation-score which is similarly smoothed. This modified Conseq-profile has minima at positions with low evolutionary diversity. Low evolutionary diversity is taken to indicate essential residues for structure or function of the protein.

I-Mutant2.0, MUpro and Conseq predictions along with the biological verified recombination sites of 1BLS are shown in Figure 1. I-Mutant2.0 and MUpro predict minima at the verified recombination sites-supporting the assumption to cut at less sensitive regions (high acceptance of substitutions). Conseq on the other hand does not seem directly applicable for the identification of recombination sites. It should be noted however that the experimental recombination data for 1BLS is far from exhaustive. Our comparison merely serves to illustrate that currently there appears to exist no silver bullet for recombination site prediction.

STAR uses continuum secondary structure as input. However, STAR does more than avoid breaking helices. Rat reductase (1AMO) has a bundle of short helices, interrupted by coiled segments (around residues 375–450). As the user of the online service can verify, STAR recognises the bundle and predicts a constant high connectivity score.

**Figure 3:**
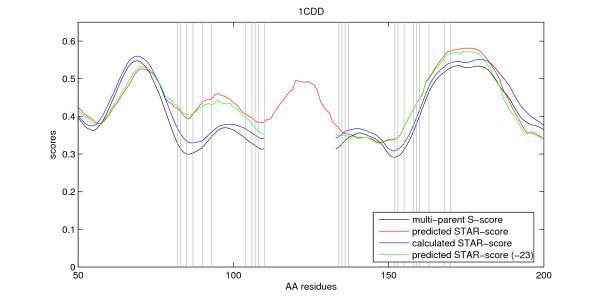
**The SCHEMA-profile and the STAR-profile for the protein **1CDD. The multi-parent S-score for 1CDD and GART (normalised) and the single-parent S-score for 1CDD (normalised), along with the predicted STAR-score for 1CDD. The gap is caused by the lack of information in the PDB file for residues 110 to 133. The prediction for the complete sequence accurately disqualifies recombination in this area, while agrees with the prediction generated for a sequence in which these 23 residues were removed. The successful recombination sites from a DNA shuffling experiment are added and plotted as vertical lines [23, 24].

PurN and GART glycinaminid ribonucleotide transformylase (70% sequence identity) were recombined and functional hybrid proteins were selected [23,24]. Recombination was restricted to occur between amino acid position 50 and 150. In Figure 3, the multi-parent S-profile determined from the PDB entry 1CDD and GART [24] is shown. The predicted STAR-profile was generated from the sequence of 1CDD. For comparison the graph also shows the calculated single-sequence S-profile for 1CDD. In spite of the high sequence similarity between 1CDD and GART, the single- and multi-parent profiles are very similar. The 1CDD structure is incomplete for residues 110–133 and hence the calculated S-profiles are undefined for this segment. The predictor was presented with the full sequence (which is known for 1CDD) and correctly characterises this segment as unsuitable for recombination. Finally, we removed all residues in the unknown sequence segment to illustrate the predictors ability to cope with incomplete sequence data.

MUpro and I-Mutant2.0 performed poorly on 1CDD (data not shown). Conseq failed completely to generate an output since 1CDD has too few family members to generate the required alignment.

## Conclusion

SCHEMA-based guidance can increase the fraction of properly folded proteins resulting from a single round of recombination from a mere 9% to 75% [8]. However, access to tertiary and quaternary structures is limited, imposing a severe restriction on the use of algorithms like SCHEMA. If exploration beyond the few sequences that have been rigorously characterised is required (e.g. to use promising products generated by an initial round of recombination), we cannot use methods that assume access to tertiary or quaternary structure.

As at April 2006, there are about 35,000 structurally resolved proteins in the Protein Data Bank but several hundreds of thousands of known proteins in sequence databases. Since STAR requires only the protein sequence as input, it enables the protein engineer to choose candidate proteins on the basis of functional properties (say, specific enzymatic activity), and not be limited to those for which full structural or extensive protein family information is available.

STAR has been trained on data generated by the normalised version of the single-parent S-profile on the basis of resolved protein structures, representative for the whole protein universe (as known through PDB). STAR is able to generalise so that each position in any hypothetical or yet unresolved protein can be accurately evaluated in relation to its potential to disrupt the structure (should it be used as a recombination site). In this context, we review recent algorithms intended for predicting structural stability changes caused by single-point mutagenesis, and adapt them to similarly serve to identify sites prone to disrupt structure.

## Availability

• Project name : STAR

• Project home page : 

• Operating System(s): Platform independent

• Programming language: Java servlet

• Licence: a licence is required for non-academic use

## Authors' contributions

DCB researched and developed the SCHEMA score prediction model under the supervision of MB and RT. DCB and MB drafted the paper and RT and

EMG provided substantive feedback on the draft and helped finishing the manuscript. The prediction service was developed by MB and DCB.
